# Immunomodulatory and Anti-Inflammatory Effects of Phycocyanin Oligopeptide Combined with Selenium and Zinc in LPS-Induced Mice

**DOI:** 10.3390/biomedicines14061309

**Published:** 2026-06-09

**Authors:** Kazim Sahin, Mehmet Yabas, Cemal Orhan, Besir Er, Muhammed F. Göktas, Nurhan Sahin, Turkkan O. Kaygusuz, Ibrahim H. Ozercan, James R. Komorowski

**Affiliations:** 1 Department of Animal Nutrition, Faculty of Veterinary Medicine, Firat University, Elazig 23119, Türkiye; corhan@firat.edu.tr (C.O.); nsahin@firat.edu.tr (N.S.); 2 Department of Immunology, Faculty of Medicine, Malatya Turgut Ozal University, Malatya 44210, Türkiye; mehmet.yabas@ozal.edu.tr; 3 Department of Medical Services and Techniques, Vocational School of Health Services, Firat University, Elazig 23119, Türkiye; ber@firat.edu.tr; 4 Department of Chest Diseases, Sincan Training and Research Hospital, Ankara 06949, Türkiye; mfrkngkts@gmail.com; 5 Department of Infectious Diseases & Clinical Microbiology, Faculty of Medicine, Firat University, Elazig 23119, Türkiye; turkkan69@firat.edu.tr; 6 Department of Pathology, Faculty of Medicine, Firat University, Elazig 23119, Türkiye; ozercanih@firat.edu.tr; 7 Research and Development, JDS Therapeutics, Harrison, NY 10577, USA; jkomorowski@jdstherapeutics.com

**Keywords:** inflammation, zinc, selenium, phycocyanin, immunomodulation, LPS

## Abstract

**Objective:** Nutritional modulation of the immune function provides a promising strategy to mitigate systemic inflammation associated with viral and bacterial infections. This study evaluated the immunomodulatory and anti-inflammatory effects of a novel phycocyanin oligopeptide (PC-O) supplement, alone or in combination with selenium (Se) and zinc (Zn), in a lipopolysaccharide (LPS)-induced murine model of inflammation. **Methods:** Forty-two male BALB/c mice were pretreated with Se, Zn, PC-O or a combination of these for 14 days, followed by LPS administration to induce systemic inflammation. Serum biochemical markers, tissue oxidative stress parameters, pro-inflammatory cytokines, antioxidant enzyme activities, histopathological alterations, Zn transporter expression, and inflammatory signaling proteins were evaluated. **Results:** LPS administration induced pronounced hepatic and pulmonary inflammation, characterized by elevated IL-1β, IL-6, and TNF-α levels, increased oxidative stress, and disrupted expression of Zn transporters. While PC-O, Se, or Zn alone partially attenuated these effects, combined supplementation produced the most substantial protective response. Notably, the combination group demonstrated significant reductions in rectal temperature, hepatic enzymes (ALT and AST), lipid peroxidation, and cytokine expression, alongside restored antioxidant enzyme activities and normalized Zn transporter levels. Protein expression analyses revealed marked suppression of NF-κB, cyclooxygenase-2 (COX-2), and inducible nitric oxide synthase (iNOS) in liver and lung tissues. **Conclusions:** Combined supplementation with PC-O, Se, and Zn provides enhanced protection against LPS-induced inflammation, likely through coordinated antioxidant and anti-inflammatory mechanisms. This nutritional strategy may help strengthen host defense and limit inflammation-driven tissue injury.

## 1. Introduction

Adequate intake of macronutrients and micronutrients plays a fundamental role in regulating immune system development and function, and is essential for maintaining immune competence and resistance to infectious diseases. Diets that meet energy and nutrient requirements support immune homeostasis. Conversely, both undernutrition and overnutrition are associated with immune dysregulation and increased susceptibility to infections [1,2]. In this context, respiratory viral and bacterial infections represent a major global health burden. They are characterized by high morbidity and mortality, and have the capacity to cause seasonal epidemics and pandemics [3,4]. Importantly, disease severity is increasingly recognized as depending not only on pathogen burden, but also on a dysregulated host immune response characterized by excessive inflammation, oxidative stress and inflammatory cell death [5].

Systemic inflammation is a key pathophysiological process that drives tissue injury associated with infection, the progression towards sepsis, multi-organ dysfunction and mortality. Excessive activation of inflammatory signaling cascades, particularly the nuclear factor kappa B (NF-κB) and mitogen-activated protein kinase (MAPK) pathways, leads to the uncontrolled production of pro-inflammatory cytokines, such as interleukin (IL)-1β, IL-6, and tumor necrosis factor-α (TNF-α). This results in hepatic and pulmonary injury, oxidative stress and metabolic dysregulation [6,7,8].

Beyond exposure to pathogens, the host’s micronutrient status, especially zinc (Zn) and selenium (Se), has emerged as a decisive modifier of immune competence and inflammatory outcomes. Deficiencies in these trace elements are common during inflammatory states and are associated with impaired immune cell function, exaggerated cytokine production, and poorer outcomes in infectious diseases and sepsis [6,9,10]. Zn plays a pivotal role in innate and adaptive immunity by regulating immune cell development, cytokine production and intracellular signaling. Inflammatory stimuli, such as lipopolysaccharide (LPS), disrupt Zn transporter expression, leading to an intracellular imbalance of Zn that amplifies oxidative stress and NF-κB-dependent inflammation [11,12]. Se exerts complementary immunomodulatory effects through selenoproteins that regulate redox balance and suppress inflammatory signaling, as demonstrated in LPS models that show reduced NF-κB/MAPK activation and lower expression of IL-6, TNF-α, COX-2, and iNOS following Se supplementation [13,14,15,16]. Similarly, phycocyanin (PC), a bioactive phycobiliprotein complex derived from Spirulina (*Arthrospira platensis*), has well-documented antioxidant, anti-inflammatory and immunomodulatory properties in hepatic, pulmonary and intestinal inflammation models, primarily by suppressing NF-κB-mediated cytokine production [17,18,19]. Importantly, controlled proteolysis of PC yields PC-O, which has enhanced antioxidant potency and improved biological activity compared with intact PC. These oligopeptides retain the chromophore–peptide architecture that underlies PC bioactivity, modulate the MAPK and Nrf2 signaling pathways, and demonstrate superior immunomodulatory efficacy in inflammation- and stress-related models [20,21,22]. Advances in solvent-free, manufacturing-compatible processing enable the scalable production of PC-O, thus supporting their potential for translation as nutraceutical immunomodulators driven by mechanisms.

From an immunopharmacological and translational perspective, combination strategies integrating micronutrients and complementary bioactive compounds are increasingly recognized as a rational means of achieving enhanced anti-inflammatory effects. These approaches simultaneously target oxidative stress, inflammatory signaling, and micronutrient-dependent immune regulation, providing more robust protection than single-agent interventions [23,24]. Notably, combined supplementation with PC, Zn, and Se has previously been shown to improve survival and reduce lung inflammation in an LPS-induced sepsis model, thus supporting the translational relevance of this strategy [8,15]. Recent studies have substantially advanced the mechanistic understanding of these bioactive nutrients in models of systemic inflammation. In murine LPS models, organic Se in the form of selenomethionine has been shown to attenuate inflammation by increasing GPX1 and selenoprotein P expression, while suppressing NF-κB/TLR4-mediated cytokine production [25,26]. Similarly, Zn supplementation has been shown to reduce NF-κB activation and the expression of COX-2 and iNOS, while contributing to the regulation of Zn transporter homeostasis [27,28]. In parallel, PC-derived peptides exhibit potent antioxidant and anti-inflammatory properties through modulation of Nrf2, Akt/AMPK, and NF-κB signaling pathways in pulmonary, intestinal, and oxidative stress-related inflammatory models [29,30,31]. Importantly, Oner et al. [8] recently demonstrated that a combination of PC oligopeptides, Zn picolinate and selenomethionine significantly improved survival and attenuated acute lung inflammation in LPS/galactosamine-challenged BALB/c mice by reducing IL-1, IL-6, TNF-α and NF-κB levels. These findings suggest that PC-O, Zn, and Se may exert complementary protective effects during endotoxin-induced inflammation. However, limited information is available regarding the combined effects of PC-O with Zn and Se on hepatic and pulmonary inflammatory responses, oxidative stress, Zn transporter regulation, and inflammatory signaling pathways in an LPS-induced systemic inflammation model. Therefore, the present study aimed to evaluate whether pre-treatment with PC-O, Zn, and Se, alone or in combination, could protect against LPS-induced systemic inflammation in mice. Particular attention was given to biochemical markers of tissue injury, oxidative stress, antioxidant defense, pro-inflammatory cytokine expression, NF-κB/COX-2/iNOS signaling, Zn transporter regulation, and histopathological alterations in liver and lung tissues. Since the supplements were administered before LPS challenge, the findings are interpreted primarily as preventive and protective rather than therapeutic effects. This approach may contribute to the development of nutritional strategies designed to strengthen host immune defenses and limit inflammation-driven tissue injury associated with infectious and inflammatory conditions.

## 2. Materials and Methods

### 2.1. Animals

Six-week-old male BALB/c mice were obtained from the Experimental Animal Center at Firat University (Elazığ, Türkiye). Throughout the study, the animals were given ad libitum access to standard rodent chow and water. The mice were housed in standard laboratory cages under controlled environmental conditions with the temperature maintained at 22 ± 2 °C, the relative humidity at 55 ± 5%, and a 12 h light/dark cycle. All experimental procedures involving the use of animals were conducted in accordance with institutional and national guidelines for the care and use of laboratory animals, and were approved by the Firat University Animal Care and Use Committee (Approval No: 15/06/2020-396351).

Food-grade PC (approximately 32% C-PC; Delhi Nutraceuticals Pvt Ltd. Haryana, India) was dissolved in demineralized water (50 g/L) with gentle stirring to prevent foam formation. The purity and composition were then checked. The quality of the PC was verified spectrophotometrically in a phosphate-buffered solution (pH 7.0) using an A620/A280 ratio of at least 1 (C-PC ≥ 30%) and an ε615 value of 5.92 mL mg^−1^ cm^−1^. The low-molecular-weight components (<10 kDa) were then removed by tangential-flow ultrafiltration (10 kDa MWCO Hydrosart), concentrating and washing the retentate until the residual <10 kDa material was <1%. The >10 kDa protein fraction was adjusted to 50 g/L and hydrolyzed with porcine pepsin (1000 FIP U/L) at 37 °C for 36 h at pH 1.2 (food-grade HCl), in a sealed vessel. Reaction progress was tracked using HPLC and A640. The digest was then neutralized to pH 7.0 (NaOH) and re-filtered (10 kDa MWCO) to collect the <10 kDa PC-O. This was freeze-dried at −70 °C and 0.42 mbar to produce powder [32]. The identity and yield of the PC-O were confirmed by photometry (A640/A280) and by HPLC (Agilent Technologies 1220 Infinity LC system, Agilent, Santa Clara, CA, USA) using an Acclaim 120 C8 column (ThermoFisher Scientific, Horsham, UK) with detection at 280 nm for peptides and at 615 nm for chromopeptides (10–16 min). The ε615 value for the PC-O (18.8 mL mg^−1^ cm^−1^) indicated a conversion of approximately 10.2% of the total PC mass [33]. Representative HPLC chromatograms revealed multiple chromopeptide peaks primarily distributed within the 10–16 min retention interval, consistent with the presence of low-molecular-weight PC-derived oligopeptides. The final PC-O fraction predominantly consisted of peptides <10 kDa generated through pepsin hydrolysis followed by tangential-flow ultrafiltration. Additional chromatographic and biochemical characterization data were provided by Komorowski [33].

### 2.2. Study Design and Treatments

A total of 42 six-week-old male BALB/c mice were randomly divided into six experimental groups of 7 mice each.

1. Normal Control: Received vehicle only (phosphate-buffered saline, PBS; Sigma, St. Louis, MO, USA).
2. LPS + Vehicle: Received vehicle, followed by LPS (Sigma, St. Louis, MO, USA) administration.
3. LPS + Se: Received Se [(200 µg human equivalent dose (HED)], supplied as selenomethionine.
4. LPS + Zn Picolinate: Received elemental Zn (30 mg HED) from Zn picolinate (20% elemental Zn).
5. LPS + PC-O: Received PC-O (300 mg HED).
6. LPS + PC-O + Zn picolinate + Se: Received a combined treatment comprising all three active compounds at the same doses used in Groups 3–5. Zn picolinate and Selenomethionine: were obtained by Nutrition 21 (New York, NY, USA).

All supplements were administered orally once daily for 14 consecutive days prior to the LPS challenge. On day 15, groups 2–6 received LPS (0.04 mg/kg, i.p.) 30 min after the final oral administration. The LPS dose used in the pyrexia model (0.04 mg/kg) was selected to induce a moderate febrile inflammatory response without causing severe endotoxemia or septic shock, consistent with previously validated low-dose LPS pyrexia models, including the protocol reported by Antwi et al. [34] LPS-induced endotoxemia is commonly used as a translational experimental model to mimic systemic inflammation associated with infection and to evaluate immunomodulatory and anti-inflammatory interventions that target the host response rather than the pathogen burden alone. Controls received 50 µL of PBS. All experimental procedures were performed between 08:00 and 09:00 GMT to minimize circadian variability. Rectal temperature was recorded at baseline and at 30 min intervals for 4 h following the LPS injection. Six hours after the administration of LPS, the mice were euthanized, and blood, liver, and lung samples were collected for biochemical, molecular, and histological analyses.

Structurally, PC consists mainly of C-phycocyanin (C-PC) and allophycocyanin (APC), which are chromoproteins containing the tetrapyrrole chromophore phycocyanobilin (PCB). C-PC forms an α/β heterodimer with covalently bound PCB, which confers its characteristic fluorescence and enables it to serve as a functional biomarker of redox and immune status [33,35].

### 2.3. Dose Calculations

Dose selection was based on the HED conversion method described by Shin et al. [36], with a human-to-mouse conversion factor of 12.33 used. The HED comprised 200 µg of Se from selenomethionine, 30 mg of Zn from Zn picolinate, and 300 mg of PC-O. The calculations are as follows:

Selenomethionine: 200 µg/70 kg = 2.86 µg/kg × 12.33 = 35.2 µg/kg/day per mouse

Zn picolinate (20% Zn): 30 mg/70 kg = 0.428 mg/kg × 12.33 = 5.28 mg/kg Zn = 26.4 mg/kg/day per mouse (Zn picolinate)

PC-O: 300 mg/70 kg = 4.29 mg/kg × 12.33 = 52.8 mg/kg/day per mouse

The selected HED values were based on clinically and experimentally established supplemental dose ranges. The 200 µg Se dose remains within the tolerable upper intake level (UL) of 400 µg/day established by the Institute of Medicine (IOM) [37] and corresponds to one of the most widely studied immunomodulatory doses used in the NPC and SELECT clinical trials reported by Clark et al. [38] and Lippman et al. [39], respectively. The 30 mg elemental Zn dose is a commonly used anti-inflammatory supplemental dose below the IOM UL of 40 mg/day [37], and Zn picolinate was selected for its superior bioavailability reported by Barrie et al. [40]. The 300 mg PC-O dose falls within the biologically active range previously reported for C-phycocyanin in experimental inflammatory models by Romay et al. [41,42]. Moreover, hydrolysis enhances the bioavailability of low-molecular-weight PC-O fractions, supporting the biological relevance of the selected dose.

### 2.4. Biochemical Analysis

Serum biochemical markers, including alanine aminotransferase (ALT), aspartate aminotransferase (AST), alkaline phosphatase (ALP), lactate dehydrogenase (LDH), blood urea nitrogen (BUN), creatinine and total bilirubin, were measured using a portable, automated chemistry analyzer (Samsung LABGEO PT10, Samsung Electronics Co., Ltd. Suwon, Republic of Korea). Lipid peroxidation was assessed by measuring malondialdehyde (MDA) levels in liver and lung using HPLC [43]. The activities of the antioxidant enzymes superoxide dismutase (SOD), catalase (CAT), and glutathione peroxidase (GSHPx) in tissue were quantified using commercially available enzyme-linked immunosorbent assay kits (Cayman Chemical, Ann Arbor, MI, USA) following the manufacturer’s protocols. Absorbance measurements were performed using an ELISA microplate reader (ELx800, BioTek Instruments Inc., Winooski, VT, USA) [44]. Mouse-specific IL-1β (KE10003; detection range: 7.8–500 pg/mL; sensitivity: 1.0 pg/mL), IL-6 (KE10007; detection range: 15.6–1000 pg/mL; sensitivity: 3.8 pg/mL), and TNF-α (KE10002; detection range: 7.8–500 pg/mL; sensitivity: 1.0 pg/mL; Proteintech Group, Rosemont, IL, USA) kits were used for the analyses. All measurements were performed in duplicate, and cytokine concentrations were calculated from standard curves generated using recombinant rat protein standards provided with each kit. To determine peripheral neutrophil counts, blood samples were collected into EDTA-coated tubes immediately before tissue collection. Neutrophil counts were measured as part of the complete blood count using an automated veterinary hematology analyzer (Exigo EOS Veterinary Hematology System; Boule Medical AB, Spånga, Sweden) according to the manufacturer’s instructions. The concentrations of Zn in liver and lung samples were determined after acid digestion. In brief, approximately 50 mg of tissue was digested with 2 mL of concentrated HNO_3_ and 1 mL of 30% H_2_O_2_ (*v*/*v*) in a microwave digestion system (Berghof Speedwave Four, Eningen, Germany) at 180–250 °C for 20 min. The digested samples were then diluted to the appropriate volume with deionized water. Zn levels were measured in triplicate using flame atomic absorption spectrometry (AAS, Perkin Elmer, Norwalk, CT, USA) with acetylene–air flame atomization at a wavelength of 213.9 nm and Zeeman background correction. Se concentrations were analyzed using graphite furnace atomic absorption spectrometry with longitudinal Zeeman background correction and an autosampler, as previously described [45,46,47]. Analytical accuracy was validated using certified reference material (bovine muscle, BCR-184). Recovery rates were 99.24% for Zn and 99.55% for Se, with respective detection limits of 0.078 mg/L and 1.68 µg/L.

### 2.5. Western Blotting Analysis

Protein expression levels of pro-inflammatory cytokines and signaling molecules, including NF-κB, TNF-α, IL-1β, IL-6, IL-17, COX-2, and iNOS, were assessed in liver and lung tissues by Western blotting, as previously described [21,46]. Additionally, proteins involved in Zn metabolism and transport were analyzed, including Zn importers (ZIP1, ZIP4 and ZIP14) and Zn exporters (ZNT1 and ZNT4). The tissues were homogenized in an ice-cold, hypotonic lysis buffer containing 50 mM Tris-HCl (pH 7.4), 150 mM NaCl, 1 mM EDTA, 1% Triton X-100, 0.1% SDS, and a protease/phosphatase inhibitor cocktail, using a glass-Teflon homogenizer. The homogenates were then centrifuged at 12,000 *g* for 15 min at 4 °C, and the resulting supernatants were collected for protein quantification using the Bradford assay. Equal amounts of protein (20 µg per lane) were separated on 10% SDS-polyacrylamide gels and transferred onto nitrocellulose membranes at 100 V for 1 h. Membranes were blocked with 5% non-fat dry milk in Tris-buffered saline containing 0.1% Tween-20 (TBST) for 1 h at room temperature. Following blocking, membranes were incubated overnight at 4 °C with the following rabbit polyclonal primary antibodies (Proteintech Group, Rosemont, IL, USA), all validated for Western blot application in mouse tissue: anti-NF-κB p65 (10745-1-AP, 1:1000), anti-TNF-α (17590-1-AP, 1:1000), anti-IL-1β (16806-1-AP, 1:1000), anti-IL-6 (21865-1-AP, 1:1000), anti-IL-17 (26163-1-AP, 1:1000), anti-COX-2 (12375-1-AP, 1:1000), anti-iNOS (18985-1-AP, 1:1000), anti-ZIP1 (20212-1-AP, 1:500), anti-ZIP4 (20625-1-AP, 1:500), anti-ZIP14 (26540-1-AP, 1:500), anti-ZNT1 (22661-1-AP, 1:500) and anti-ZNT4/SLC30A4 (24828-1-AP, 1:500). All primary antibodies were used at the dilutions specified above and were validated by the respective manufacturers for Western blot application in mouse tissue, with specificity confirmed by the detection of a single immunoreactive band at the predicted molecular weight. Following primary antibody incubation, membranes were washed three times with TBST (10 min each) and then incubated for 1 h at room temperature with HRP-conjugated goat anti-rabbit IgG secondary antibody (SA00001-2, 1:5000). Protein bands were visualized using an enhanced chemiluminescence (ECL) detection system and quantified by densitometric analysis using NIH ImageJ software (version 1.53, National Institutes of Health, Bethesda, MD, USA). To ensure equal protein loading, all membranes were stripped and reprobed with a rabbit polyclonal anti-β-actin antibody (cat. no. 20536-1-AP, Proteintech; 1:2000) as a housekeeping reference, and the relative expression of each target protein was normalized to the corresponding β-actin signal.

### 2.6. Histological Analysis

Liver and lung tissues were fixed in 10% neutral-buffered formalin, embedded in paraffin and processed for histological analysis [48,49]. Paraffin sections (3 µm thick) were deparaffinized, rehydrated and stained with hematoxylin and eosin (H&E). Histopathological changes and inflammatory alterations were examined under a light microscope. Histopathological evaluations were performed in a blinded manner by an experienced histopathologist unaware of the treatment groups. Liver sections were semi-quantitatively scored for hepatocellular degeneration, sinusoidal congestion, inflammatory cell infiltration, and tissue architecture disruption, whereas lung sections were evaluated for alveolar wall thickening, edema, inflammatory infiltration, and structural damage. Histopathological alterations were graded using a semi-quantitative scoring system as follows: 0 = absent, 1 = mild, 2 = moderate, and 3 = severe tissue injury.

### 2.7. Statistical Analysis

A power analysis determined that a sample size of seven mice per group would provide 85% power to detect significant differences at a significance level of *p* < 0.05. Statistical analyses were conducted using SPSS software (IBM SPSS Version 22.0). Prior to statistical comparisons, the normality of data distribution was evaluated using the Shapiro–Wilk test, and homogeneity of variances was assessed using Levene’s test. Since the data satisfied the assumptions of normality and homogeneity, group comparisons were conducted using one-way analysis of variance (ANOVA), followed by Tukey’s post hoc test for multiple comparisons. Differences were considered statistically significant at *p* < 0.05. In figures and tables, different lowercase letters (a–e) indicate statistically significant differences among groups, whereas groups sharing at least one common letter are not significantly different.

## 3. Results

### 3.1. Thermal Response to LPS and Protective Effects of Interventions

**Figure 1:**
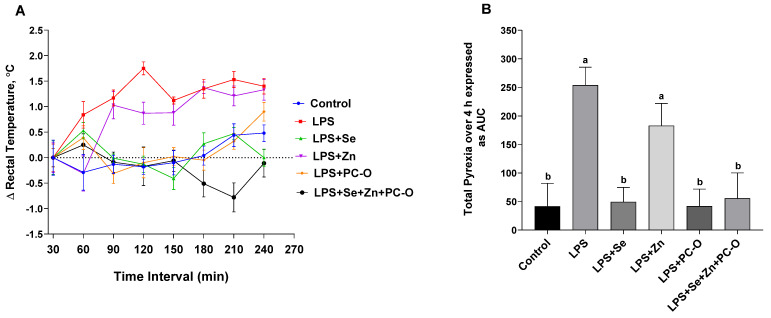
The effects of Se, Zn and PC-O on the LPS-induced febrile response in mice. (**A**) Time course of rectal temperature measurements following LPS administration, showing the febrile response across treatment groups. (**B**) Total pyrexia, quantified as the area under the temperature-time curve (AUC). Mice were pretreated with Se, Zn, PC-O or a combination of these prior to the LPS challenge. Each symbol represents an individual mouse. Data were analyzed using one-way ANOVA followed by Tukey’s post hoc test. Different lowercase letters (a, b) indicate statistically significant differences among groups (*p* < 0.05). Groups sharing at least one common letter are not significantly different.

Pyrexia was assessed by measuring rectal temperature, and the temporal progression is depicted in Figure 1A. Both the LPS and LPS + Zn groups exhibited significant pyrexia. In contrast, the LPS + Se, LPS + PC-O and LPS + Se + Zn + PC-O groups showed temperature profiles similar to those of the control group. Figure 1B shows the overall febrile response, quantified as the thermal index (TI), which is calculated as the area under the temperature-time curve (AUC) over 4 h. The peak rectal temperature and AUC were observed in the LPS group. Notably, LPS-induced pyrexia was significantly reduced in the LPS + Se, LPS + PC-O and LPS + Se + Zn + PC-O groups (Figure 1).

### 3.2. Biochemical Markers of Hepatic and Renal Function Following LPS-Induced Inflammation and Protective Effect

Table 1 summarizes serum biochemical parameters reflecting hepatic and renal function. Significant intergroup variations were detected for ALT, AST, ALP, LDH, BUN, creatinine and total bilirubin. LPS administration resulted in a marked increase in ALT activity compared with the control group. Pre-treatment with Se, Zn, PC-O, or a combination of these significantly attenuated this increase, with the most pronounced reduction observed in the Se + Zn + PC-O group. However, levels remained above control values. AST levels followed a similar trend, with the highest value in the LPS group compared with the control group. Pre-treatment with Se or Zn alone moderately reduced AST levels, with further decreases observed in the LPS + PC-O and LPS + Se + Zn + PC-O groups. The combined supplementation group exhibited the most substantial reduction. ALP activity was also significantly increased in the LPS group. The lowest ALP levels were recorded in the LPS + Se + Zn + PC-O group and differed significantly from those in the LPS + Se and LPS + PC-O groups.

LDH activity peaked in the LPS group, whereas all treatment groups showed significantly lower levels (Table 1). The greatest reduction was again observed in the LPS + Se + Zn + PC-O group, indicating an improved protective effect. Similar effects were observed for renal function markers. BUN levels were significantly elevated in the LPS group compared to the control group. Although no significant differences were detected among them, all treatment groups showed reductions in BUN. Serum creatinine levels increased significantly following LPS administration compared to the control group (Table 1). Supplementation with PC-O and a combination regimen (LPS + Se + Zn + PC-O) both reduced creatinine levels, with the latter group approaching control values most closely. Total bilirubin levels were markedly higher in the LPS group than in the control group. Supplementation with Se, Zn, PC-O, or a combination of these led to progressive reductions in bilirubin levels, with the LPS + Se + Zn + PC-O group showing the greatest decrease (Table 1).

### 3.3. Oxidative Stress Markers and Antioxidant Enzyme Activities in Liver and Lung Tissues

The oxidative stress parameters and antioxidant enzyme activities in the liver and lungs were then evaluated and summarized in Table 2. Significant differences were observed among the groups in MDA levels and the activities of SOD, CAT, and GSHPx. In the liver, MDA levels were lowest in the control group, but increased significantly following LPS administration. The supplementation groups (LPS + Se, LPS + Zn, LPS + PC-O, and LPS + Se + Zn + PC-O) showed a progressive reduction in MDA levels, with the combination regimen (LPS + Se + Zn + PC-O) demonstrating the greatest decrease (Table 2). Conversely, hepatic SOD activity was highest in the control group and was significantly reduced in the LPS group. Partial restoration of SOD activity was noted across the treatment groups, with the greatest recovery observed in the LPS + Se + Zn + PC-O group (Table 2).

Similar trends were seen for CAT activity, which reduced significantly following LPS administration but increased with supplementation, peaking in the combination group (Table 2). GSHPx activity followed a similar pattern: it was highest in the control group, decreased by LPS and was partially restored by supplementation. The most substantial increase was seen in the LPS + Se + Zn + PC-O group (Table 2). In lung tissue, MDA levels were significantly elevated in the LPS group than in the control group, but decreased progressively in the supplementation groups, reaching the lowest concentration in the combination group (Table 2). SOD, CAT and GSHPx activities in the lung mirrored the liver results, showing significant reductions after LPS exposure and notable recovery in the treatment groups, particularly in the LPS + Se + Zn + PC-O group (Table 2).

### 3.4. Levels of Serum Pro-Inflammatory Cytokines and Neutrophil Response Following LPS and Preventive Interventions

**Figure 2:**
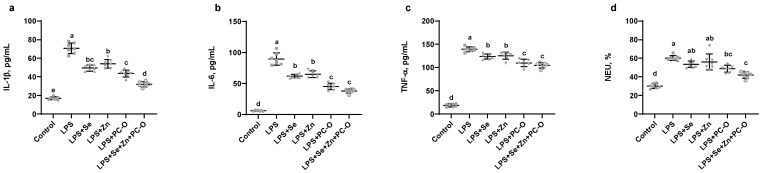
The effects of Se, Zn and PC-O on systemic inflammatory markers in LPS-induced inflammation in mice. Serum levels of IL-1β (**a**), IL-6 (**b**), TNF-α (**c**) and neutrophil (NEU) count (**d**) were measured to assess systemic inflammation following the administration of LPS and subsequent treatments. Each symbol represents an individual mouse. Different letters (a–e) indicate statistically significant differences among groups (*p* < 0.05) as determined by one-way ANOVA followed by Tukey’s post hoc test. Groups sharing at least one common letter are not significantly different.

We subsequently evaluated the levels of key pro-inflammatory cytokines to determine the inflammatory response induced by LPS and the modulatory effects of the administered treatments. As shown in Figure 2, LPS administration resulted in significant increases in the pro-inflammatory cytokines IL-1β (A), IL-6 (B) TNF-α (C), as well as in neutrophil (NEU) counts (D). Notably, IL-1β concentrations in the LPS + Se, LPS + Zn and LPS + PC-O groups were comparable, indicating that these individual supplementation exerted similar modulatory effects. The combination regimen (LPS + Se + Zn + PC-O) resulted in the most significant reduction in IL-1β, suggesting an enhanced anti-inflammatory effect (Figure 2a). Similarly, the LPS + Se and LPS + Zn groups exhibited intermediate reductions in IL-6, whereas the LPS + PC-O group and the combination pre-treatment significantly attenuated IL-6 expression, indicating enhanced efficacy in dampening inflammation (Figure 2b). Supplementation with Se or Zn alone did not produce significant differences in TNF-α levels. However, the LPS + PC-O and LPS + Se + Zn + PC-O groups showed the lowest TNF-α expression, highlighting their anti-inflammatory potential (Figure 2c). Additionally, while no significant differences were observed among the LPS + Se, LPS + Zn and LPS + PC-O groups, the combined pre-treatment group (LPS + Se + Zn + PC-O) demonstrated the greatest reduction in NEU counts, second only to the control group. This indicates a significant improvement in inflammation control (Figure 2d).

### 3.5. Assessment of Zn and Se Concentrations in the Liver and Lung Tissues of Mice

**Figure 3:**
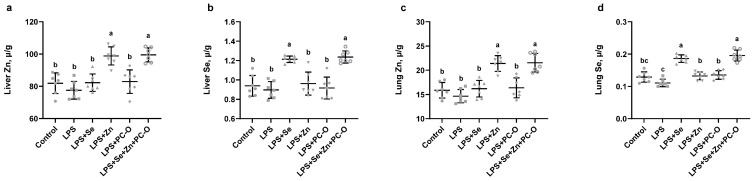
Tissue concentrations of Zn and Se following treatment with Se, Zn and PC-O in a LPS-induced pyrexia model in mice. Quantification of Zn and Se levels in liver (**a**,**b**) and lung (**c**,**d**) tissues across experimental groups. Mice were pretreated with Se, Zn, PC-O or a combination of these prior to LPS administration. Each data point symbol represents an individual animal. Different letters (a–c) above the data points indicate statistically significant differences among groups (*p* < 0.05) as determined by one-way ANOVA followed by Tukey’s post hoc test. Groups sharing at least one common letter are not significantly different from each other.

Systemic inflammation in the present study was induced in mice by the intraperitoneal administration of LPS. Zn and Se concentrations were first quantified in liver and lung tissues, and the results are presented in Figure 3. Liver Zn levels were comparable across the control, LPS, LPS + Se, and LPS + PC-O groups, showing no statistically significant differences observed. However, significant elevations in hepatic Zn were observed in the LPS + Zn and LPS + Se + Zn + PC-O groups, consistent with Zn supplementation (Figure 3a). Similarly, liver Se concentrations did not differ significantly between the control, LPS, LPS + Zn, and LPS + PC-O groups. In contrast, Se levels were significantly higher in the LPS + Se and LPS + Se + Zn + PC-O groups, reflecting effective Se supplementation (Figure 3b). In lung tissue, Zn concentrations remained low and were statistically indistinguishable among the control, LPS, LPS + Se, and LPS + PC-O groups. Marked significant increases in lung Zn were detected in the LPS + Zn and LPS + Se + Zn + PC-O groups, which differed significantly from the other groups (Figure 3c). Lung Se levels followed a similar pattern, with no significant variation between the control, LPS, LPS + Zn and LPS + PC-O groups. Conversely, elevated Se concentrations were found in the LPS + Se and LPS + Se + Zn + PC-O groups, showing significant differences relative to the other groups (Figure 3d).

### 3.6. Modulation of Pro-Inflammatory Cytokines in Liver and Lung Tissues

**Figure 4:**
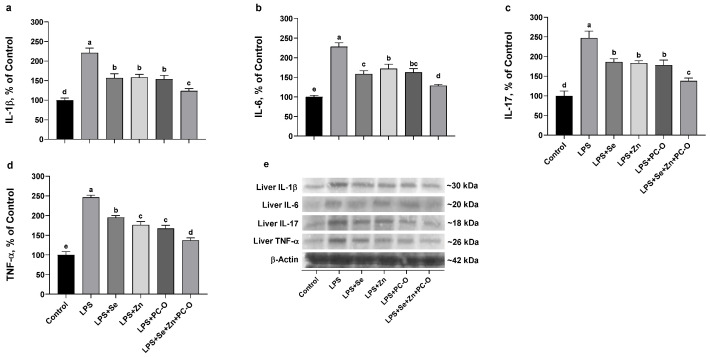
The effect of Se, Zn and PC-O on hepatic IL-1β (**a**), IL-6 (**b**), IL-17 (**c**) and TNF-α (**d**) protein expression in LPS-induced inflammation. Western blot analysis was used to quantify protein expression, with densitometric analysis normalized to β-actin to ensure equal protein loading. Data are presented as a percentage of the control group, which was set to 100%. Representative images (**e**) are shown. Data were analyzed using one-way ANOVA followed by Tukey’s post hoc test. Molecular weight (M.W.) markers are indicated in kilodaltons (kDa). Different lowercase letters (a–e) indicate statistically significant differences among groups (*p* < 0.05). Groups sharing at least one common letter are not significantly different.

**Figure 5:**
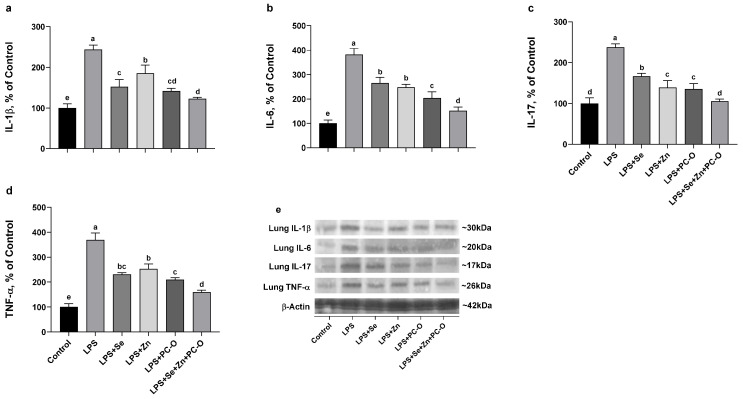
The effect of Se, Zn and PC-O on the protein levels of IL-1β (**a**), IL-6 (**b**), IL-17 (**c**) and TNF-α (**d**) in the lung of mice with LPS-induced pyrexia. Western blot analysis was used to quantify protein expression, with densitometric analysis normalized to β-actin to ensure equal protein loading. Data are presented as a percentage of the control group, which was set to 100%. Representative images (**e**) are shown. Data were analyzed using one-way ANOVA followed by Tukey’s post hoc test. Molecular weight (M.W.) markers are indicated in kilodaltons (kDa). Different lowercase letters (a–e) indicate statistically significant differences among groups (*p* < 0.05). Groups sharing at least one common letter are not significantly different.

To corroborate these findings, the levels of pro-inflammatory cytokines in liver and lung tissues were evaluated by Western blotting (Figure 4 and Figure 5). IL-1β expression varied significantly across the experimental groups. Compared to the control group, LPS treatment substantially increased IL-1β levels, indicating a pronounced inflammatory response (Figure 4a). Supplementation with Se, Zn or PC-O partially attenuated this elevation. Notably, the combined pre-treatment (LPS + Se + Zn + PC-O) resulted in the lowest IL-1β expression, suggesting an enhanced protective effect of the combined supplementation. Similarly, liver IL-6 levels were elevated significantly following LPS administration, and all supplementation regimens reduced IL-6 expression. The most marked decrease was observed in the combination group (Figure 4b). IL-17 levels also showed significant intergroup differences. LPS exposure increased IL-17 expression, whereas supplementation with Se, Zn and PC-O mitigated this response. The combined regimen exhibited the strongest reduction (Figure 4c). TNF-α expression followed a comparable pattern. Compared to the control group, LPS increased TNF-α levels, and the treatment groups displayed decreased TNF-α expression. The combined group demonstrated the most substantial decrease, highlighting the enhanced efficacy of the integrated treatment approach (Figure 4d).

Key inflammatory mediators were also quantified in lung tissue by Western blot analysis (Figure 5). Lung IL-1β levels increased markedly following LPS administration. Supplementation with Se, Zn, and PC-O each partially mitigated this elevation. The greatest reduction was observed in the combination group (LPS + Se + Zn + PC-O) (Figure 5a). A similar pattern was observed for IL-6: it was upregulated in the LPS group; however, supplementation with Se, Zn, and PC-O reduced IL-6 levels. The combination regimen again showed the most substantial effect, reducing IL-6 expression (Figure 5b). Exposure to LPS elevated IL-17 expression, but individual treatments with Se, Zn and PC-O moderated this increase. The LPS + Se + Zn + PC-O group reduced IL-17 expression to near-baseline levels (Figure 5c). Similarly, Se and Zn supplementation reduced LPS-induced TNF-α expression. The combination pre-treatment yielded the greatest anti-inflammatory effect (Figure 5d). Taken together, these results confirm that LPS induces robust pro-inflammatory cytokine expression in the lung, which can be significantly attenuated by Se, Zn and PC-O supplementation, particularly when combined, suggesting an enhanced protective effect against LPS-induced inflammation.

### 3.7. Expression of Inflammatory Signaling Targets in Liver and Lung Tissues

**Figure 6:**
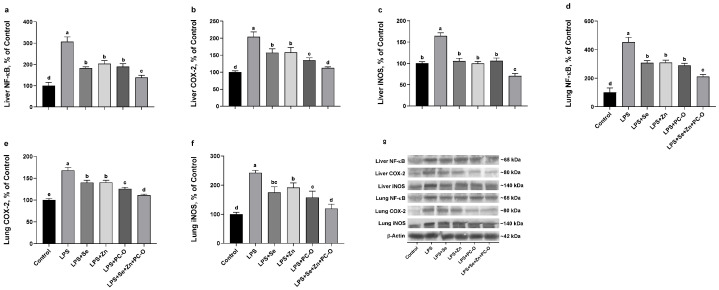
The effect of Se, Zn and PC-O on the protein levels of NF-κβ, COX-2 and iNOS in liver (**a**–**c**) and lung (**d**–**f**) tissues of mice with LPS-induced pyrexia. Western blot analysis was used to quantify protein expression, with densitometric analysis normalized to β-actin to ensure equal protein loading. Data are presented as a percentage of the control group, which was set to 100%. Representative images (**g**) are shown. Data were analyzed using one-way ANOVA followed by Tukey’s post hoc test. Molecular weight (M.W.) markers are indicated in kilodaltons (kDa). Different lowercase letters (a–e) indicate statistically significant differences among groups (*p* < 0.05). Groups sharing at least one common letter are not significantly different.

In order to investigate the impact of various treatments on key inflammatory signaling pathways, the expression levels of NF-κB, COX-2, and iNOS were quantified in liver and lung tissues. In the liver, LPS administration resulted in a significant increase in NF-κB protein levels. Co-administration of Se or Zn with LPS reduced NF-κB expression, and administration with LPS, Se, Zn and PC-O suppressed it further (Figure 6a). Similarly, COX-2 expression was increased in response to LPS alone. This increase was mitigated in the LPS + Se, LPS + Zn and LPS + PC-O groups. The most notable attenuation was observed in the LPS + Se + Zn + PC-O group (Figure 6b). iNOS expression in the liver increased following LPS exposure, but this effect was diminished by Se, Zn or PC-O supplementation. The LPS + Se + Zn + PC-O group exhibited a more substantial reduction (Figure 6c).

A comparable pattern was observed in lung tissue. LPS markedly elevated NF-κB expression, which was reduced in the LPS + Se, LPS + Zn and LPS + PC-O groups. Supplementation with Se, Zn and PC-O further reduced NF-κB expression (Figure 6d). COX-2 levels in the lung increased following LPS treatment. Supplementation with Se or Zn resulted in slightly lower levels, while PC-O led to a more modest increase. COX-2 expression was significantly reduced in the combination group (Figure 6e). iNOS expression in the lung increased with LPS treatment. However, the Se, Zn and PC-O groups showed reduced iNOS expression compared to the LPS group. Interestingly, the combination supplementation effectively reduced iNOS levels to a level similar to the control (Figure 6f). Collectively, these findings suggest that pre-treatment with Se, Zn, and PC-O may attenuate the LPS-induced upregulation of pro-inflammatory mediators in both liver and lung tissues, potentially modulating key inflammatory pathways.

### 3.8. Effects of LPS and Treatments on Expression of Zn Transporters in Liver and Lung Tissues

**Figure 7:**
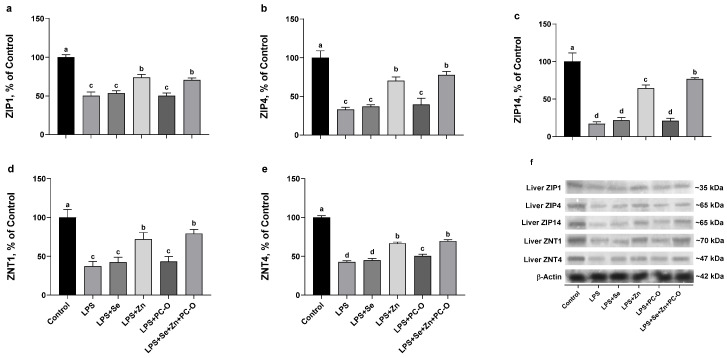
The effect of Se, Zn and PC-O on liver protein levels of ZIP1 (**a**), ZIP4 (**b**), ZIP14 (**c**) and ZNT1 (**d**) and ZNT4 (**e**) in mice with LPS-induced pyrexia. Western blot analysis was used to quantify protein expression, with densitometric analysis normalized to β-actin to ensure equal protein loading. Data are presented as a percentage of the control group, which was set to 100%. Representative images (**f**) are shown. Data were analyzed using one-way ANOVA followed by Tukey’s post hoc test. Molecular weight (M.W.) markers are indicated in kilodaltons (kDa). Different lowercase letters (a–d) indicate statistically significant differences among groups (*p* < 0.05). Groups sharing at least one common letter are not significantly different.

To assess the impact of LPS and various supplementations on Zn homeostasis, the levels of several Zn transporters (ZIP1, ZIP4, ZIP14, ZNT1 and ZNT4) were evaluated in the liver and lung via Western blot analysis (Figure 7a–e). LPS reduced ZIP1 expression in the liver. The LPS + Se group showed a modest increase, while the LPS + Zn administration elevated expression. PC-O alone had no effect, but the LPS + Se + Zn + PC-O group restored ZIP1 expression (Figure 7a). LPS exposure significantly downregulated ZIP4 expression, and Se and PC-O supplementation provided only minimal improvement. Zn alone, however, restored ZIP4 expression. Combined pre-treatment further increased ZIP4 expression (Figure 7b). A comparable trend was observed for ZIP14, which decreased markedly after LPS treatment. Se and PC-O supplementation resulted in minimal, non-significant increases, while Zn treatment significantly restored its expression. The LPS + Se + Zn + PC-O combination further improved ZIP14 expression (Figure 7c). ZNT1 expression was also significantly suppressed by LPS. Se and PC-O alone did not significantly reverse this effect, but Zn supplementation restored levels. Combined supplementation further enhanced its expression (Figure 7d).

**Figure 8:**
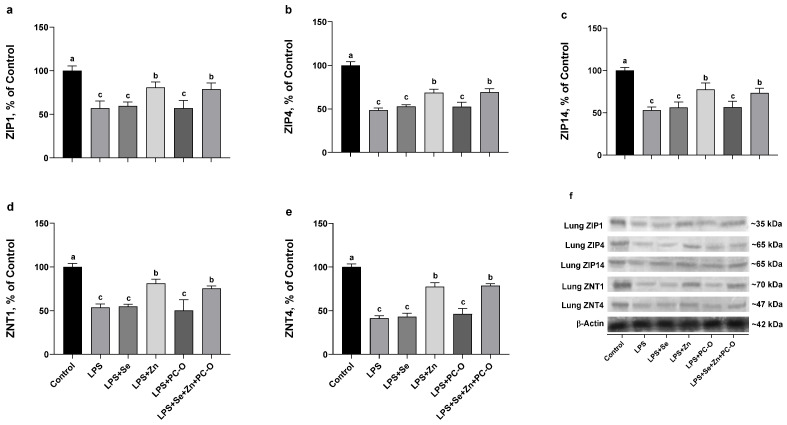
The effect of Se, Zn and PC-O on lung protein levels ZIP1 (**a**), ZIP4 (**b**), ZIP14 (**c**), ZNT1 (**d**) and ZNT4 (**e**) in mice with LPS-induced pyrexia. Western blot analysis was used to quantify protein expression, with densitometric analysis normalized to β-actin to ensure equal protein loading. Data are presented as a percentage of the control group, which was set to 100%. Representative images (**f**) are shown. Data were analyzed using one-way ANOVA followed by Tukey’s post hoc test. Molecular weight (M.W.) markers are indicated in kilodaltons (kDa). Different lowercase letters (a–c) indicate statistically significant differences among groups (*p* < 0.05). Groups sharing at least one common letter are not significantly different.

LPS treatment reduced lung ZIP1 expression (Figure 8a). Neither Se nor PC-O had a significant effect on ZIP1 expression, whereas Zn significantly increased it. A similar effect was observed with the combination treatment, which restored ZIP1 expression (Figure 8b). ZIP4 expression declined significantly after LPS treatment. Both the Se and PC-O groups showed partial restoration, and Zn supplementation increased their expression. Combined supplementation further improved the restoration of ZIP4 levels in the lung (Figure 8c). LPS also suppressed ZIP14 expression. The Se and PC-O groups maintained their expression levels, while Zn supplementation significantly increased ZIP14 expression. The combination group exhibited a similar recovery (Figure 8d). ZNT1 expression in the lung was reduced by LPS treatment. The Se and PC-O groups exhibited low expression, whereas Zn treatment increased expression. The combination regimen increased ZNT1 expression (Figure 8e). Finally, ZNT4 expression decreased following LPS exposure. Se and PC-O co-treatments showed limited improvement, whereas Zn supplementation significantly restored expression. The LPS + Se + Zn + PC-O combination yielded a level comparable to that of the control (Figure 8f). Overall, these results suggest that LPS disrupts Zn homeostasis and transporter expression in liver and lung tissues, while combined supplementation with Se, Zn, and PC-O effectively mitigates these alterations, likely through modulation of regulatory pathways.

### 3.9. Histopathological Evaluation of Liver and Lung Tissues Following LPS Administration and Treatment Interventions

**Figure 9:**
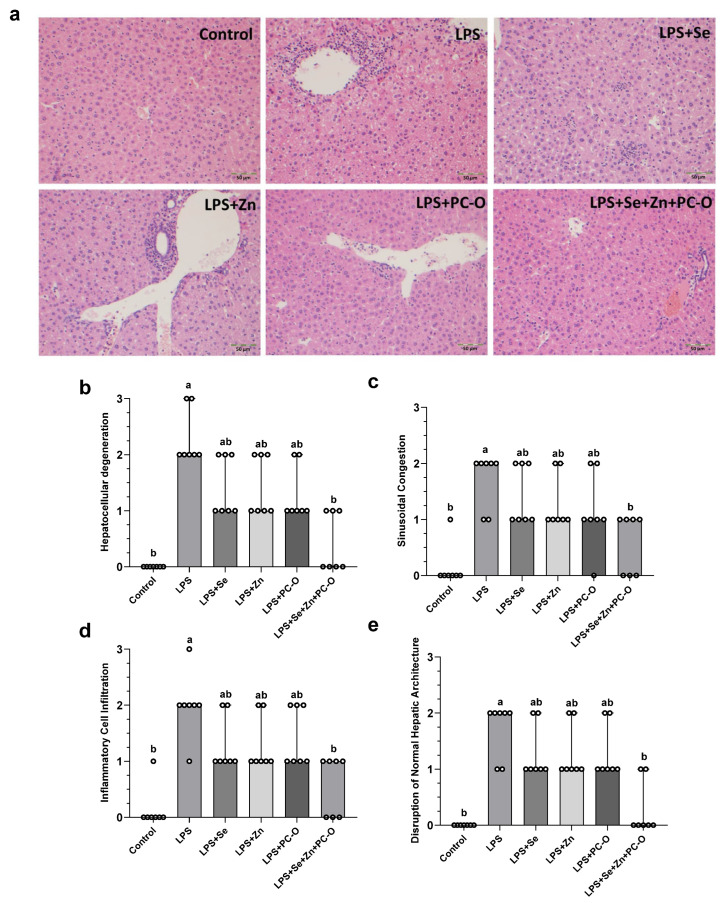
Histopathological analysis (H&E) of liver section from the Control, LPS, LPS + Se, LPS + Zn, LPS + PC-O, and LPS + Se + Zn + PC-O groups (200× magnification) (**a**). Histopathological alterations were evaluated semi-quantitatively in a blinded manner. Semi-quantitative histopathological scoring of hepatocellular degeneration (**b**), sinusoidal congestion (**c**), inflammatory cell infiltration (**d**), and disruption of normal hepatic architecture (**e**). Histopathological lesions were graded on a scale of 0–3 (0 = absent, 1 = mild, 2 = moderate, 3 = severe). Data are presented as median values with individual data points. Different letters (a, b) indicate statistically significant differences among groups according to the Kruskal–Wallis test followed by Dunn’s multiple comparison post hoc test (*p* < 0.05).

Histopathological evaluation of H&E-stained liver sections demonstrated marked hepatic injury in the LPS-treated group compared with the control group (Figure 9a). LPS administration induced severe hepatocellular degeneration, sinusoidal congestion, inflammatory cell infiltration, and disruption of normal hepatic architecture, confirming the development of acute inflammatory liver damage. Semi-quantitative histopathological scoring further supported these findings, showing significantly increased scores for hepatocellular degeneration, sinusoidal congestion, inflammatory cell infiltration, and disruption of normal hepatic architecture in the LPS group compared with controls (Figure 9b–e). Supplementation with Se, Zn, or PC-O alone partially ameliorated the LPS-induced histopathological alterations, as evidenced by reduced inflammatory infiltration and moderate preservation of hepatic structure. Although the individual treatments decreased the severity of hepatocellular degeneration and sinusoidal congestion, histological scores remained higher than those of the control group. In contrast, the Se + Zn + PC-O supplementation produced the greatest hepatoprotective effect, markedly attenuating inflammatory cell infiltration, congestion, and hepatocellular degeneration while preserving the overall hepatic architecture. Histopathological scores in the combined treatment group were significantly lower than those observed in the LPS group and approached control values, indicating substantial protection against LPS-induced hepatic injury (Figure 9b–e).

**Figure 10:**
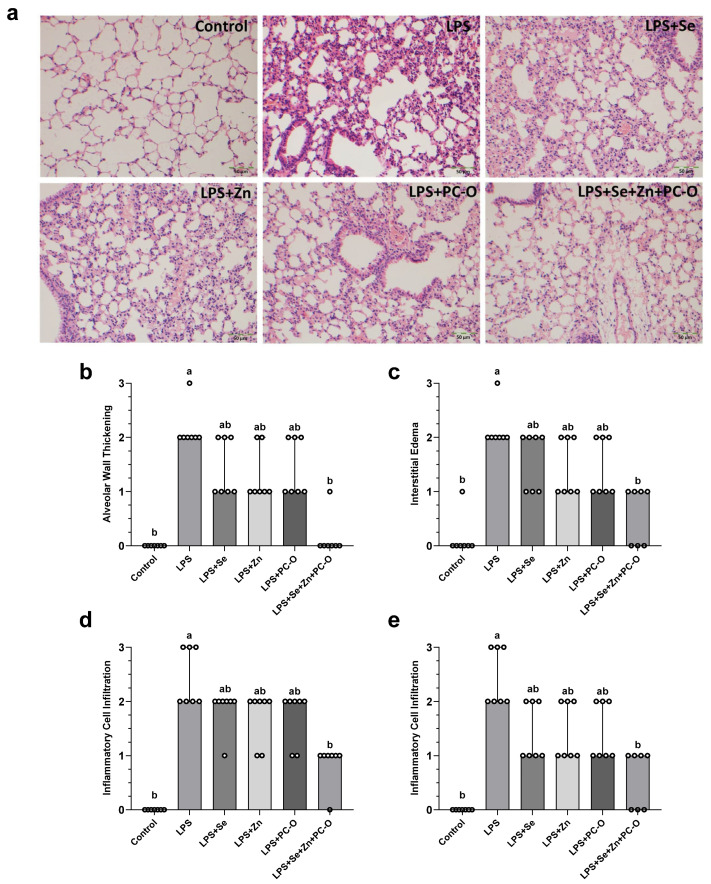
Representative hematoxylin and eosin (H&E)-stained lung tissue sections from the Control, LPS, LPS + Se, LPS + Zn, LPS + PC-O, and LPS + Se + Zn + PC-O groups (original magnification, ×200) (**a**) Histopathological alterations were evaluated semi-quantitatively in a blinded manner. Semi-quantitative histopathological scoring of alveolar wall thickening (**b**), interstitial edema (**c**), inflammatory cell infiltration (**d**), and architectural disarray (**e**). Histopathological lesions were graded on a scale of 0–3 (0 = absent, 1 = mild, 2 = moderate, 3 = severe). Data are presented as median values with individual data points. Different letters indicate statistically significant differences among groups according to the Kruskal–Wallis test followed by Dunn’s multiple comparison post hoc test (*p* < 0.05).

Histopathological examination of H&E-stained lung tissues demonstrated severe pulmonary damage in the LPS-treated group compared with the control group (Figure 10a). LPS exposure caused marked alveolar wall thickening, interstitial edema, inflammatory cell infiltration, and disruption of normal pulmonary architecture, indicating acute inflammatory lung injury. Semi-quantitative histopathological scoring confirmed significant increases in alveolar wall thickening, interstitial edema, inflammatory cell infiltration, and overall inflammatory cell accumulation in the LPS group relative to controls (Figure 10b–e). Administration of Se, Zn, or PC-O alone moderately attenuated the LPS-induced histopathological alterations, as evidenced by reduced edema, decreased inflammatory cell infiltration, and partial preservation of alveolar structure. Nevertheless, histopathological scores in these treatment groups remained higher than those of the control group. In contrast, supplementation with Se + Zn + PC-O exhibited the strongest protective effect against LPS-induced pulmonary injury. Lung tissues from this group showed nearly normal alveolar morphology, minimal inflammatory cell infiltration, reduced pulmonary congestion, and markedly improved tissue organization. Correspondingly, histopathological scores were significantly lower than those observed in the LPS group and approached control values, indicating substantial protection against acute inflammatory lung damage (Figure 10b–e).

## 4. Discussion

This study investigated the preventive and protective effects of Se, Zn and PC-O, administered either individually or in combination in a murine model of LPS-induced systemic inflammation. The results show that combined pre-treatment with Se, Zn, and PC-O attenuated LPS-induced inflammatory and oxidative responses more effectively than the individual components. This protection was associated with improved hepatic and pulmonary architecture, reduced biochemical markers of tissue injury, restoration of antioxidant enzyme activities, and partial normalization of Zn transporter expression. These findings suggest that this combined nutritional approach may strengthen host resilience against endotoxin-induced inflammatory stress.

The innate immune system acts as the body’s first line of defense against bacterial infections. It orchestrates a complex response involving immune cells, cytokines, and pro-inflammatory mediators to eliminate pathogens [34,50]. Although activation of the innate immune system is essential for clearing pathogens, an excessive or prolonged inflammatory response, such as sustained pyrexia, can result in tissue damage to the host and systemic complications [51,52]. Dysregulated immune activation, as observed in sepsis, can result in widespread injury to vital organs, including the liver, lungs and circulatory system [53,54]. These outcomes highlight the importance of immune homeostasis in maintaining host integrity during infection. LPS, a prototypical pathogen-associated molecular pattern, triggers inflammation by activating pattern-recognition receptors such as TLR4, thereby inducing downstream cascades of NF-κB, cytokine release, oxidative stress and cellular injury [55,56]. Previous studies on LPS-induced inflammation have demonstrated that certain bioactive compounds can modulate immune responses and mitigate systemic damage [34,55]. In our study, we found that LPS administration caused a pronounced febrile response, as evidenced by significant increases in rectal temperature and thermal index (TI). This effect was attenuated in mice pretreated with Se, PC-O, or their combination with Zn, while Zn alone provided limited protection. The most notable reduction in pyrexia was observed in the Se + Zn + PC-O group, whose temperature profiles were similar to those of the control animals. These results imply that both Se and PC-O have potent antipyretic and immunomodulatory properties, and that Zn enhances this protection. These results are consistent with previous evidence indicating that Se supports adaptive immune responses and reduces excessive cytokine release [57,58], while PC and its derivatives inhibit pro-inflammatory signaling and oxidative pathways [59,60]. Together, they appear to act through complementary mechanisms that stabilize thermo-regulatory and immune responses during acute inflammation.

It is known that inflammation alters the metabolism and tissue distribution of trace elements. During acute-phase responses, Zn and Se are often redistributed to the liver, leading to decreased peripheral availability and impaired enzyme activity [61,62]. Supplementation effectively corrected these deficiencies in a tissue-specific manner. Zn supplementation alone or in combination significantly increased Zn concentrations in both organs, while Se and combined treatments restored Se levels. These results confirm the efficient absorption and bioavailability of both trace elements and suggest that Se and PC-O do not interfere with Zn uptake. Restoring the balance of trace elements is particularly important because Zn and Se act as cofactors for antioxidant enzymes, such as SOD and GSHPx, which are crucial in limiting oxidative injury during infection.

LPS administration produced severe biochemical disturbances, consistent with hepatic and renal injury. Serum levels of ALT, AST, ALP, LDH, BUN, creatinine and total bilirubin were markedly elevated in the LPS group, indicating hepatocellular necrosis, mitochondrial dysfunction and impaired renal filtration. While all treatment groups demonstrated biochemical improvement, the most pronounced recovery was observed in the Se + Zn + PC-O group. Transaminase normalization indicates stabilization of the hepatocellular membrane and reduced leakage of intracellular enzymes. Similarly, the reduction in LDH and bilirubin levels suggests reduced cytotoxicity and improved hepatic clearance. Improvement in BUN and creatinine levels also indicates preserved renal function. These findings support previous reports that Se enhances hepatocellular antioxidant defense [27], Zn maintains membrane integrity [63], and PC exerts hepatoprotective effects via downregulation of pro-oxidant signaling [17,60]. At the level of systemic inflammation, LPS predictably elevated IL-1β, IL-6, TNF-α, and NEU counts. The combinatorial regimen markedly suppressed these mediators, reflecting a broadly immunomodulatory effect. Suppression of NF-κB, COX-2 and iNOS in both the liver and lung further supports the notion that these agents interfere with canonical inflammatory signaling. Similar anti-inflammatory actions of PC and trace minerals have been observed across diverse models [64,65,66], and our results extend these findings within a Se + Zn + PC-framework. The present findings are further supported by a growing body of recent evidence demonstrating the anti-inflammatory properties of PC-derived peptides and their combinations with micronutrients. PC peptides have been shown to inhibit pulmonary fibrosis through HO-1/Nrf2 induction [30], reduce LPS-induced intestinal inflammation via Akt/AMPK signaling [29], and protect against oxidative tissue injury by suppressing COX-2 and TNF-α expression [67], mechanisms that are consistent with the hepatic and pulmonary protective effects observed in the present study. The superior efficacy of the PC-O + Zn + Se combination treatment is consistent with recent reports demonstrating the enhanced antioxidant and anti-inflammatory interactions between Zn and Se in multiple in vivo models [27,28]. Moreover, organic selenomethionine has recently been shown to suppress NF-κB-mediated inflammatory responses and enhance selenoprotein-dependent antioxidant defense under LPS stimulation [25,68], consistent with the reduced NF-κB and iNOS expression observed in the current study. Importantly, Oner et al. [8] previously demonstrated that combined supplementation with PC, Zn and Se significantly improved survival and attenuated acute lung inflammation in LPS/galactosamine-challenged BALB/c mice. One particularly novel aspect of this study is the regulation of Zn homeostasis under inflammatory stress. LPS exposure suppressed the expression of Zn transporter proteins, including ZIP1, ZIP4, ZIP14, ZNT1, and ZNT4. These changes may reflect inflammation-induced disruption of Zn redistribution and transporter regulation. Zn supplementation, particularly when combined with Se and PC-O, restored several of these transporter-related changes. This suggests that the combined supplementation may help preserve Zn-handling mechanisms during endotoxin-induced inflammation. The restoration of ZIP and ZNT protein expression suggests that the intervention acts by supplying Zn and by preserving the machinery for intracellular Zn trafficking. Zn plays recognized roles in redox signaling and immune regulation [62], so restoring its function likely contributes to the anti-inflammatory and cytoprotective effects observed. Mechanistically, the combination of Se, Zn and PC-O may stem from complementary pathways: (i) Se acts as a key cofactor in selenoenzymes (e.g., glutathione peroxidases), helping to scavenge reactive oxygen species; (ii) Zn stabilizes protein structure and modulates signaling enzymes and transcription factors (including NF-κB); and (iii) PC provides direct antioxidant and anti-inflammatory effects at the transcriptional and enzymatic levels.

Previous studies have demonstrated that individual micronutrients, such as Zn or Se, can reduce inflammatory injury and oxidative stress in sepsis, acute lung injury and related inflammatory conditions. This is largely achieved by modulating NF-κB and Nrf2 signaling and antioxidant enzyme activity [53,69,70,71]. Zn supplementation has been shown to reduce NEU recruitment and lung damage in LPS-induced acute lung injury, while also being associated with changes in Zn transporter-mediated homeostasis [69,71]. Similarly, Se, particularly in organic forms, has been shown to protect against LPS- or toxin-induced tissue injury by enhancing GSHPx activity, limiting oxidative stress and modulating MAPK- and NF-κB-driven inflammatory responses [14,70,72,73,74]. However, most previous studies have focused on single micronutrients or pharmacological nanoformulations, rarely examining trace elements in combination with protein-derived bioactive peptides. To our knowledge, no prior LPS model has simultaneously evaluated Se, Zn, and PC-O, alongside parallel assessment of Zn transporter regulation in both the liver and the lung, systemic cytokine profiles, pyrexia, histopathology, and canonical inflammatory signaling. Therefore, the present findings extend current research by demonstrating that combined Se + Zn + PC-O supplementation provides broader and more robust protection than the individual components across functional, structural, and molecular endpoints. They also identify the preservation of Zn-handling machinery as a potential mechanistic node linking micronutrient status to cytokine control and organ resilience during endotoxin-induced inflammatory stress [27,53,72,75].

Histological evaluations confirmed the biochemical results at a structural level, and were consistent with the histoprotective properties of trace element supplementation observed in other models of LPS-induced injury [8,64].

## 5. Limitations and Future Directions

Several limitations of the present study should be acknowledged. Firstly, the findings are based on an acute LPS-induced murine model of systemic inflammation. While this model is highly relevant for studying endotoxin-driven immune responses, it does not fully capture the complexity and chronicity of human inflammatory or infectious diseases. Additionally, only a single dose and pre-treatment duration were evaluated for PC-O, Zn and Se, preventing a detailed assessment of dose–response relationships, long-term safety and sustained efficacy. Furthermore, although key inflammatory mediators, antioxidant enzymes and Zn transporter proteins were analyzed, upstream regulatory events and immune cell-specific mechanisms were not examined directly. Tissue Zn and Se concentrations were also measured at a single time point, which limits insight into their dynamic redistribution during inflammation and recovery.

Future studies should therefore focus on elucidating the molecular basis of the observed enhancement, paying particular attention to NF-κB/MAPK signaling, Nrf2-driven antioxidant pathways, and Zn transporter regulation in specific immune and parenchymal cell populations. Time-course and dose-optimization studies will be necessary to define optimal supplementation strategies under both acute and chronic inflammatory conditions. Extending this approach to disease-relevant models of chronic low-grade inflammation, infection-associated organ injury and metabolic-inflammatory disorders would further strengthen the translational relevance of this research. Finally, investigations into formulation, bioavailability and safety will be essential for advancing this combined intervention towards nutraceutical or adjunct nutritional applications targeting inflammation-driven pathologies.

## 6. Conclusions

In conclusion, this study demonstrates that combined supplementation with Se, Zn and PC-O provides robust protection against LPS-induced systemic inflammation in mice. Pre-treatment with this regimen effectively reduces pyrexia, restores hepatic and pulmonary Zn and Se levels, and normalizes key biochemical markers of hepatic and renal injury. Marked reductions in lipid peroxidation, recovery of antioxidant enzyme activities and substantial histological improvement in liver and lung architecture supported these systemic benefits. At the molecular level, the combined treatment downregulated NF-κB-mediated inflammatory signaling, reduced the expression of COX-2, iNOS and pro-inflammatory cytokines (IL-1β, IL-6 and TNF-α), and corrected LPS-induced disturbances in the expression of Zn transporters. Compared with individual supplementation, the Se + Zn + PC-O combination produced the most pronounced protective effects, indicating a clear combined interaction between trace elements and bioactive peptides. Collectively, these findings identify a multi-targeted nutritional strategy that simultaneously supports trace element balance, antioxidant defense, and inflammatory resolution. Future research is needed to validate these effects in chronic inflammation models and to explore their clinical relevance.

## Figures and Tables

**Table 1:** The modulatory effects of Se, Zn and PC-O on the serum biochemistry of mice with LPS-induced pyrexia.

	Groups					
	Control	LPS	LPS + Se	LPS + Zn	LPS + PC-O	LPS + Se + Zn + PC-O
ALT, (IU/L)	41.57 ± 10.061 ^e^	365.29 ± 16.93 ^a^	261.57 ± 16.16 ^bc^	280.43 ± 8.85 ^b^	244.43 ± 16.37 ^c^	220.29 ± 18.74 ^d^
AST, (IU/L)	52.29 ± 5.56 ^e^	779.29 ± 59.38 ^a^	573.43 ± 59.23 ^b^	580.86 ± 32.61 ^b^	517.57 ± 38.20 ^bc^	458.00 ± 50.36 ^c^
ALP, (IU/L)	59.86 ± 15.04 ^d^	318.43 ± 31.63 ^a^	285.43 ± 10.33 ^b^	292.71 ± 15.34 ^ab^	266.71 ± 12.65 ^bc^	243.14 ± 9.82 ^c^
LDH, (IU/L)	66.09 ± 5.84 ^e^	277.59 ± 16.03 ^a^	192.11 ± 10.72 ^c^	216.13 ± 11.39 ^b^	189.83 ± 5.39 ^c^	170.41 ± 6.71 ^d^
BUN, (mg/dL)	19.46 ± 1.50 ^c^	40.87 ± 4.92 ^a^	34.36 ± 3.26 ^b^	35.57 ± 3.20 ^ab^	32.44 ± 2.89 ^b^	32.03 ± 3.64 ^b^
Creatinine, (mg/dL)	0.42 ± 0.06 ^c^	0.70 ± 0.07 ^a^	0.59 ± 0.08 ^ab^	0.59 ± 0.06 ^ab^	0.55 ± 0.06 ^b^	0.52 ± 0.09 ^bc^
T Bilirubin, (mg/dL)	0.29 ± 0.06 ^d^	1.20 ± 0.12 ^a^	0.95 ± 0.08 ^b^	0.97 ± 0.08 ^b^	0.90 ± 0.05 ^bc^	0.82 ± 0.04 ^c^

Data were analyzed using one-way ANOVA followed by Tukey’s post hoc test. Data are presented as the mean ± SD. Different lowercase letters (a–e) indicate statistically significant differences among groups (*p* < 0.05). Groups sharing at least one common letter are not significantly different.

**Table 2:** The impact of the Se, Zn and PC-O on the levels of antioxidant enzymes in the liver and lung of mice with LPS induced pyrexia.

	Groups					
	Control	LPS	LPS + Se	LPS + Zn	LPS + PC-O	LPS + Se + Zn + PC-O
Liver, MDA, (nmol/mg)	3.24 ± 0.22 ^e^	7.11 ± 0.46 ^a^	5.86 ± 0.26 ^b^	5.92 ± 0.35 ^b^	5.16 ± 0.34 ^c^	4.34 ± 0.36 ^d^
Liver, SOD, (U/mg)	153.7 ± 9.11 ^a^	56.37 ± 5.29 ^d^	95.16 ± 5.06 ^b^	75.94 ± 5.93 ^c^	77.19 ± 7.29 ^c^	103.35 ± 6.74 ^b^
Liver, CAT, (U/mg)	86.89 ± 5.45 ^a^	31.49 ± 2.57 ^d^	41.89 ± 4.03 ^c^	43.06 ± 3.24 ^c^	42.03 ± 3.26 ^c^	60.71 ± 3.54 ^b^
Liver, GSHPx, (U/mg)	61.32 ± 3.77 ^a^	21.39 ± 1.96 ^e^	44.79 ± 3.86 ^c^	36.86 ± 1.98 ^d^	37.98 ± 2.16 ^d^	49.64 ± 3.56 ^b^
Lung, MDA (nmol/mg)	4.18 ± 0.44 ^d^	9.24 ± 0.42 ^a^	6.67 ± 0.49 ^b^	6.83 ± 0.30 ^b^	6.69 ± 0.37 ^b^	5.89 ± 0.37 ^c^
Lung, SOD, (U/mg)	105.78 ± 6.72 ^a^	31.89 ± 2.86 ^d^	59.32 ± 4.81 ^b^	50.36 ± 2.93 ^c^	49.28 ± 5.04 ^c^	65.75 ± 3.93 ^b^
Lung, CAT, (U/mg)	52.30 ± 3.70 ^a^	18.69 ± 1.43 ^d^	32.29 ± 1.86 ^b^	28.53 ± 1.70 ^c^	27.76 ± 2.76 ^c^	35.12 ± 1.75 ^b^
Lung, GSHPx, (U/mg)	46.15 ± 3.97 ^a^	16.86 ± 1.42 ^d^	33.86 ± 3.41 ^b^	28.08 ± 1.98 ^c^	26.63 ± 2.46 ^c^	36.75 ± 2.53 ^b^

Data were analyzed using one-way ANOVA followed by Tukey’s post hoc test. Different lowercase letters (a–e) indicate statistically significant differences among groups (*p* < 0.05). Groups sharing at least one common letter are not significantly different.

## Data Availability

The datasets generated and/or analyzed in this study are available from the corresponding author upon reasonable request. During the preparation of this manuscript, the authors used ChatGPT (OpenAI, GPT-5.5, accessed in 2026) for language editing and to improve the clarity and readability of the text. The authors reviewed and edited the content as needed and take full responsibility for the content of the published article.

## Abbreviations

The following abbreviations are used in this manuscript:

IL	Interleukin
LPS	Lipopolysaccharide
PC-O	Phycocyanin oligopeptide
Se	Selenium
Zn	Zinc
